# IL-17 Exerts Anti-Apoptotic Effect via miR-155-5p Downregulation in Experimental Autoimmune Encephalomyelitis

**DOI:** 10.1007/s12031-017-0981-2

**Published:** 2017-10-23

**Authors:** Dominika Ksiazek-Winiarek, Piotr Szpakowski, Malgorzata Turniak, Janusz Szemraj, Andrzej Glabinski

**Affiliations:** 1 Department of Neurology and Stroke, Medical University of Lodz, Zeromskiego 113 Street, 90-549 Lodz, Poland; 2 Department of Medical Biochemistry, Medical University of Lodz, Mazowiecka 6/8 Street, 92-215 Lodz, Poland

**Keywords:** microRNAs, Multiple sclerosis, Interleukin-17, Experimental autoimmune encephalomyelitis, Bcl-2

## Abstract

Multiple sclerosis is an autoimmune, neurodegenerative disease, affecting mostly young adults and resulting in progressive disability. It is a multifactorial disorder, with important involvement of both cellular and epigenetic components. Among the epigenetic factors, microRNAs are currently intensively investigated in the context of multiple sclerosis. It has been shown that their biogenesis and function may be regulated by various cytokines. IL-17, a hallmark cytokine of Th17 cells, has been thought to function predominantly as a pro-inflammatory factor, leading to increased disease symptoms. However, there are several studies indicating its protective role during inflammatory process. In this work, we have assessed the impact of high-dose IL-17 administration on microRNAs’ expression profile during the preclinical stage of EAE. For selected microRNA, we have performed computational analysis of its potential target mRNAs and cellular pathways. Based on results obtained from in silico analysis, we have chosen genes from neurotrophin signaling pathway for further experiments—*BDNF*, *HRAS*, and *BCL2*. Results obtained in this study suggested that high dose of IL-17 exerts protective activity via miR-155-5p downregulation. Increased expression of all studied genes, especially *BCL2*, indicated a potential anti-apoptotic function of IL-17 during the preclinical phase of EAE.

## Introduction

Multiple sclerosis (MS) is a chronic autoimmune disease of the central nervous system (CNS) characterized by the presence of inflammatory infiltrates, demyelination, and axonal degeneration, resulting in progressive neurological disability (Trapp et al. 1999). Autoreactive Th1 and Th17 cells are considered as crucial players in the MS development. These T cell subpopulations can initiate and sustain inflammatory reaction in the CNS, leading to irreversible tissue damage observed in MS patients (Compston and Coles 2008). Furthermore, the Th1 to Th17 ratio is thought to affect the CNS lesion load and disease severity (Stromnes et al. 2008).

Th17 cells are relatively recently discovered subtype of T helper cells. They boost inflammatory response in the context of pathogens’ invasion and autoimmunity (Littman and Rudensky 2010). IL-17 is the hallmark cytokine produced by the Th17 cells. It is generally accepted that IL-17 is a pro-inflammatory molecule. It increases the production of chemokines, leading to enhanced recruitment of monocytes and neutrophils to the site of inflammation (Spriggs 1997; Witowski et al. 2000). Moreover, IL-17 intensifies production of inflammatory cytokines by macrophages (Ng et al. 2011). It also drives T cell responses, partially via induction of ICAM expression (Albanesi et al. 1999). It was observed that in MS patients, the level of IL-17 was increased both in brain lesions and mononuclear cells isolated from blood and cerebrospinal fluids (Lock et al. 2002; Matusevicius et al. 1999). However, in the last few years, several studies have presented evidence that in specific conditions, IL-17 may exert protective function (Hamour et al. 2015; Hu et al. 2014; Ke et al. 2009).

Despite numerous studies, the pathogenesis of multiple sclerosis is still not fully unrevealed. It is suggested that environmental and epigenetic factors may play important role in MS development. microRNAs (miRNAs) are one of the newly discovered epigenetic regulators. They are small non-coding RNA molecules (about 21–25 nucleotides long) regulating mRNA translation. miRNAs function via the RISC (RNA-induced silencing complex), through the binding to their complementary sequence located in the 3′UTR of targeted mRNA (Bartel 2009). microRNAs have been shown to modulate various aspects of immunity, from regulation of cells development, to their activation and function (Dai and Ahmed 2011). Moreover, inflammatory cytokines and microRNAs can be regulated reciprocally (Junker 2011). Altered expression and function of these molecules may result in development of autoimmune disease, like MS.

In recent years, numerous studies have indicated the important role of various microRNAs in the pathogenesis of MS, and its animal model—experimental autoimmune encephalomyelitis (EAE) (Cox et al. 2010; Junker et al. 2009; Ma et al. 2014; O’Connell et al. 2010a). miR-155 is one of the best characterized miR. Its role in different aspects of disease has been widely confirmed. miR-155 participates in blood-brain barrier (BBB) breakdown and enhances differentiation and activation of Th1 and Th17 cells—the major effector cells for MS and EAE development and progression (Lopez-Ramirez et al. 2014; O’Connell et al. 2010a; Stromnes et al. 2008). It also regulates function of CNS resident cells, resulting in enhanced neuroinflammation and neurodegeneration (Cardoso et al. 2012; Junker et al. 2009).

The aim of our study was to analyze the impact of IL-17 administration on microRNA expression profile in the initial stage of EAE. Moreover, we want to analyze the functional relevance of such deregulation for neurotrophin signaling pathway, which is important for EAE and MS pathogenesis.

## Materials and Methods

### Animals

**Fig. 1:**
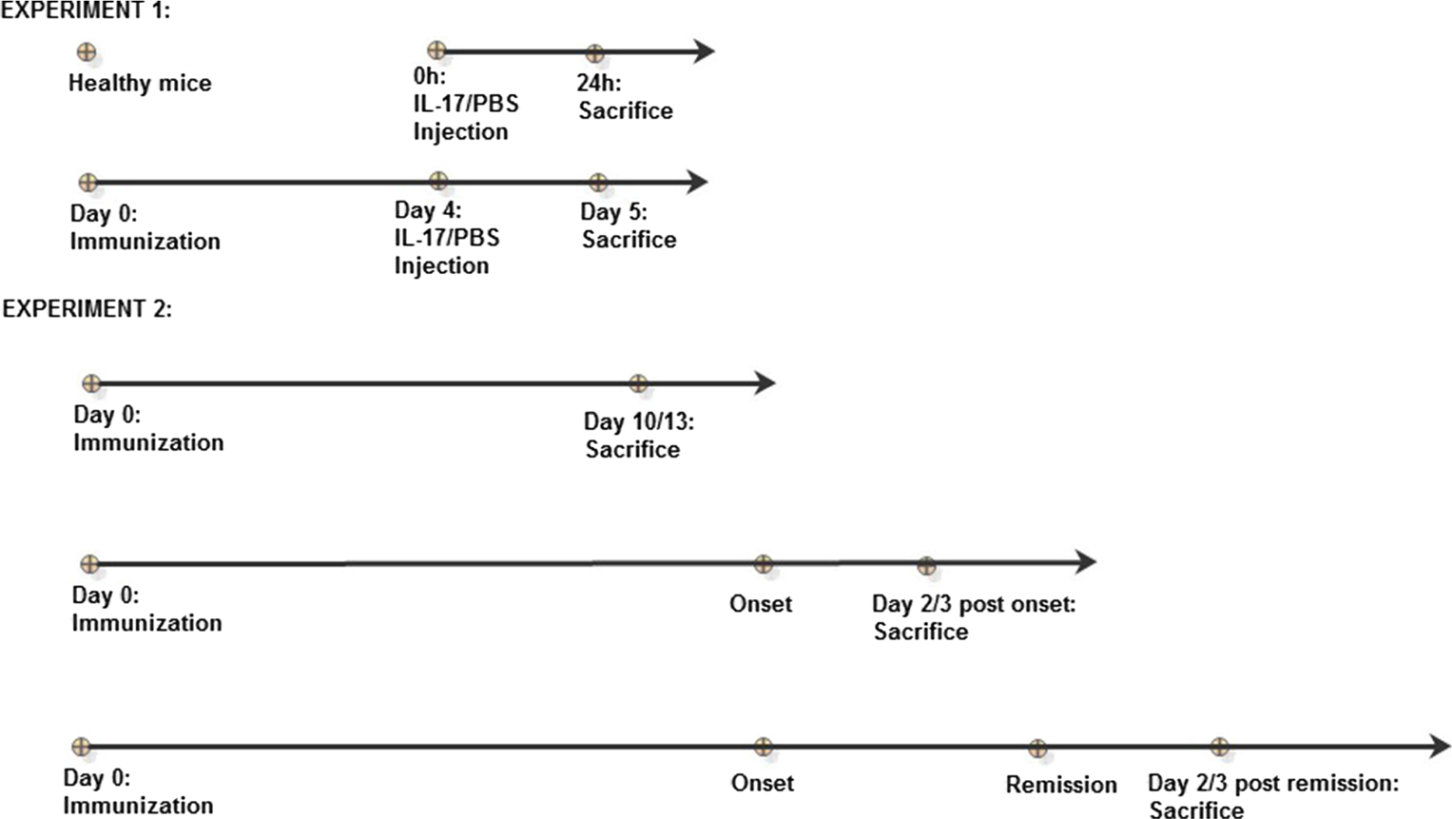
The mice were used in two independent experiments. In the first, one healthy animal and immunized mice were subjected to stereotactic brain microinjection of IL-17 or PBS (phosphate-buffered saline) (*n* = 10 per group). In the second experiment, immunized mice were sacrificed in different stages of disease (*n* = 10 per group)

Eight-to-twelve-week-old female SJL mice were purchased from Charles River Laboratories (Sulzfeld, Germany). During all experiments, mice were maintained in SPF conditions in the animal facility of Medical University of Lodz. All procedures were approved by the Local Ethics Committee at the Medical University of Lodz. The experiments’ design is presented on the scheme below (Fig. 1).

### Induction of EAE

EAE was induced by subcutaneous injection of proteolipid protein (PLP) 139-151 peptide (Metabion, Martinsried, Germany) on both sides near the axillary lymph nodes. Prior to injection, PLP 139-151 was emulsified with complete Freund’s adjuvant (Sigma, Poznan, Poland). *Bordetella pertussis* toxin (Sigma, Poznan, Poland) was administered through tail vein injections on the day of immunization and 48 h later, as previously described (Glabinski et al. 1997). Animals were weighted and monitored daily to assess the clinical signs of EAE. The clinical scoring scale used in research was: 0—no disease symptoms; 1—decreased tail tone or slightly clumsy gait; 2—tail atony and/or moderately clumsy gait and/or poor righting ability; 3—limb weakness; 4—limb paralysis; 5—moribund state (Glabinski et al. 1997).

### Stereotactic Brain Microinjections

Stereotactic microinjections were conducted on ketamine/xylazine anesthetized mice (Biowet, Pulawy, Poland) on the fourth day post immunization (early preclinical phase) or on healthy mice. Animals were given 100 ng of IL-17A (R&D Systems, Minneapolis, MN) or PBS (CytoGen GmbH, Sinn, Germany) as a control. The procedure was performed on a stereotactic frame (David Kopf Instruments, Tujunga, CA) using a Hamilton syringe (32G needle, 0.25 mm). Injections were made into the striatum of the brain (in a volume of 1 μl), which did not cause any apparent neurological impairment in the animals. After intracerebral cytokine administration, the scalp was sutured with surgical thread. Mice were sacrificed 24 h post microinjections and brains were isolated for further RNA or protein analysis.

### Tissue Collection

Mice brains were isolated from all animals, both healthy and immunized (5DPI), subjected to stereotactic microinjections in the first experiment. In the second experiment (see Fig. 1), brains were harvested from control animals and from immunized mice during three disease stages: in preclinical phase (10–13 days post immunization, before any disease symptoms were visible); during clinical sings (at second day of relapse); and during remission (second day of disease amelioration). As a control, healthy non-immunized mice were used. Mice were anesthetized with a ketamine/xylazine mixture administered intraperitoneally and perfused through the left cardiac ventricle with the ice cold PBS (CytoGen GmbH, Sinn, Germany). Brains were collected and further processed as described below.

### RNA Isolation and Reverse Transcription PCR

Brains obtained from mice were immediately homogenized in QIAzol reagent (Qiagen, Hilden, Germany) using a homogenizer (Ultra-Turrax T8, Staufen, Germany). Total RNA enriched for short RNA molecules (from 18 nt upwards) was isolated with the miRNeasy Mini Kit (Qiagen, Hilden, Germany) according to the manufacturer’s protocol. Concentration of RNA samples was analyzed on Synergy HT Multi-Mode Microplate Reader (BioTek, Winooski, VT, USA). Samples were stored at − 80 °C until further processing.

For cDNA synthesis from RNA, the miScript II RT Kit (Qiagen, Hilden, Germany) was used according to the manufacturer’s instruction. Briefly, for mature microRNA analysis, we used 200 ng of RNA and miScript HiSpec Buffer. For mRNA reactions, we used 200 ng of RNA and miScript HiFlex Buffer. The reaction conditions were as follows: incubation for 60 min at 37 °C; incubation for 5 min at 95 °C for reverse transcriptase inactivation. The RT PCR was conducted on T100 thermal cycler (Bio-Rad, Hercules, CA, USA).

### miRNA Array and Quantitative Real-Time PCR

To analyze changes in microRNA expression profile in preclinical mice subjected to intracerebral injections of IL-17 or PBS (control), we used Mouse Inflammatory Response and Autoimmunity miScript miRNA Array (Qiagen, Hilden, Germany) according to the given protocol. A total of 84 microRNAs were analyzed. The list of the microRNAs from the array is available on the company’s web site (www.qiagen.com). First, the total RNA was transcribed to cDNA (as described earlier), then the adequate amount of cDNA and qPCR reaction mix was added across PCR Array. The cycling conditions were as follows: initial activation step—15 min at 95 °C, followed by 40 cycles of denaturation for 15 s at 94 °C, annealing step for 30 s at 55 °C, and extension for 30 s at 70 °C. For results analysis, we used free data analysis software for miScript miRNA PCR Array (http://pcrdataanalysis.sabiosciences.com/mirna).

Analysis of individual miRNAs was performed with specific miScript Primer Assays (for miR-302d-3p; miR-291a-3p; miR-694; miR-144-3p; miR-155-5p; miR-130a-3p; and miR-497a-5p) and miScript SYBR Green PCR Kit (all from Qiagen, Hilden, Germany). As a reference gene, we used SNORD95 (Qiagen). Briefly, 2 ng of cDNA was added to individual well of the PCR plate and qPCR reaction mix was dispensed into each well. The reaction conditions were as follows: initial activation step—15 min at 95 °C, followed by 40 cycles of denaturation for 15 s at 94 °C, annealing step for 35 s at 55 °C, and extension for 35 s at 70 °C.

Analysis of mRNA expression level was performed using specific QuantiTect Primer Assay (for brain-derived neurotrophic factor (*BDNF*), Harvey rat sarcoma viral oncogene homolog (*HRAS*), *BCL2*) and QuantiTect SYBR Green PCR Kit (all from Qiagen, Hilden, Germany). As a reference gene, we used Rpl13a (Qiagen). Briefly, 10 ng of cDNA was added to individual well of the PCR plate and qPCR reaction mix was dispensed into each well. The reaction conditions were as follows: initial activation step—15 min at 95 °C, followed by 40 cycles of denaturation for 15 s at 94 °C, annealing step for 30 s at 55 °C, and extension for 30 s at 72 °C.

All real-time PCR analyses were conducted on Mx3005P qPCR System (Agilent Technologies, CA, USA). All reactions were done in triplicate.

Calculations of relative expression values for RNAs were performed using the ∆∆CT method. Obtained results were presented as a fold change (2^−∆∆CT^). Results higher than fold change + 2 or lower than fold change − 2 were considered significant for miRs, as these molecules are known to possess one or more binding sites in their target mRNAs. This may result in considerable mRNA deregulation, even when miR expression level is altered moderately.

### Computational Analysis of miRNA Target Genes

To identify potential altered pathways and mRNA targets for the differentially expressed miRNA, we used online databases, such as TagetScan Mouse (Release 7.1), DIANA mirPath (v3.0) and miRecords (last updated on April 27, 2013) (Agarwal et al. 2015; Vlachos et al. 2015; Xiao et al. 2009).

### Protein Isolation and Analysis with Enzyme-Linked Immunosorbent Assay

Brains obtained from mice were homogenized in PBS (CytoGen GmbH, Sinn, Germany) with protease inhibitors cocktail (Complete Mini, Roche Diagnostics GmbH, Mannheim, Germany) using a homogenizer (Ultra-Turrax T8, Staufen, Germany). Samples were then centrifuged (20,000×*g*, 30 min, 4 °C), and supernatants were collected and diluted when needed. For proteins’ expression level analysis, commercially available ELISA kits were used according to manufacturers’ instructions (BDNF—R&D Systems, Minneapolis, MN; HRas—Wuhan EIAab Science, Wuhan, China; Bcl-2—Abbexa, Cambridge, UK). The results were normalized to the total protein concentration in our samples measured by BCA protein assay (Pierce Biotechnology, Rockford, IL). All reactions were conducted in duplicate.

### Statistical Analysis

Statistical analysis was performed using unpaired Student’s *t* test or Mann-Whitney *U* test, followed by Tukey’s HSD (honestly significant difference) posthoc analysis. Analysis of correlation between microRNA-155-5p expression level and expression of selected mRNAs was performed with Pearson’s test or Spearman’s test, according to the normal distribution. Data normality was verified with Shapiro-Wilk test. Statistical significance level was set at *p* < 0.05. Values are presented as mean ± SD where indicated. Statistical analysis was performed using Statistica v.12 software.

## Results

### High Dose of IL-17 Deregulate microRNAs’ Expression in Preclinical Phase of EAE

**Fig. 2:**
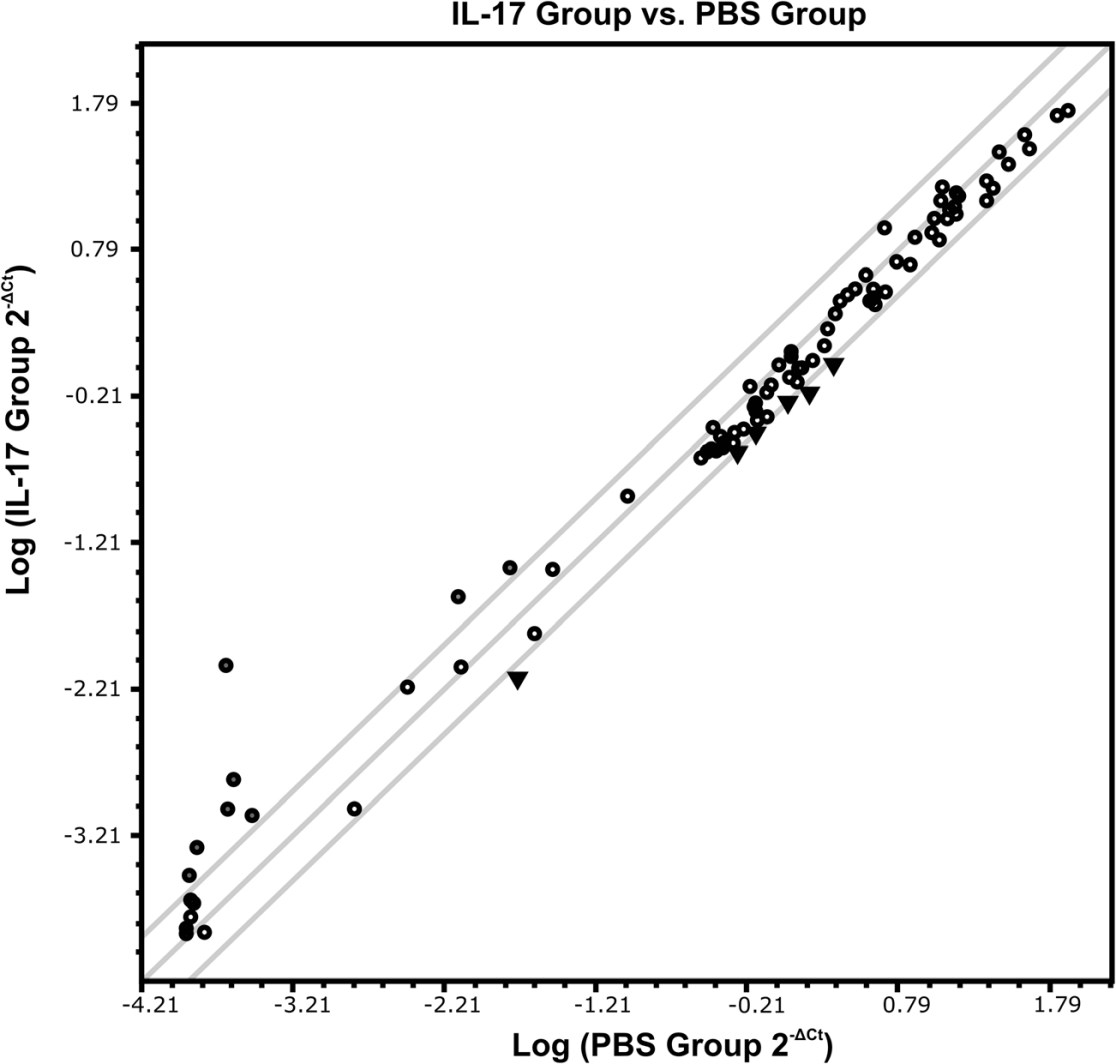
Scatterplot of relative expression of 84 microRNAs from Mouse Inflammatory Response and Autoimmunity microRNA PCR array, analyzed in brain homogenates of mice in preclinical phase of EAE, after stereotactic microinjections of IL-17 or PBS (control) (see the “Materials and Methods” section). Gray lines in the scatterplot indicate + 2.0 and – 2.0 fold change, which were used as cutoff values for selection of candidate miRNAs (black circles represent upregulated microRNAs, black triangles represent downregulated microRNAs)

To initially select microRNAs with expression levels influenced by the high dose of IL-17, we utilized pathway-focused array. We chose the Mouse Inflammatory Response and Autoimmunity (miScript miRNA Array) as these are two main processes present both in EAE and MS. Here, we used RNA isolated from brains of mice in preclinical stage of disease (day 5 post immunization), as the presence of immune cells has been reported in this time point (Wojkowska et al. 2014). Mice were subjected to brain microinjections of 100 ng IL-17 or PBS, as a control (*n* = 4 for each group). We have analyzed a total of 84 miRs. From these, 14 miRs were significantly deregulated in our experimental conditions (Fig. 2, Table 1). As the purpose of our study was to analyze the impact of IL-17 on expression level of miRs relevant for EAE development and progression, we have selected seven of the most altered miRs for further analysis during the course of disease (four upregulated and three downregulated molecules).

**Table 1:** microRNAs related to the process of inflammation and autoimmunity deregulated by intracerebral microinjections of IL-17 in preclinical phase of EAE in mice

microRNA symbol	Fold change
mmu-miR-302d-3p	39.6814
mmu-miR-291a-3p	5.8685
mmu-miR-694	4.0889
mmu-miR-294-3p	3.5393
mmu-miR-144-3p	3.488
mmu-miR-743a-3p	2.5852
mmu-miR-295-3p	2.4726
mmu-miR-712-5p	2.4609
mmu-miR-155-5p	− 2.6595
mmu-miR-130a-3p	− 2.5364
mmu-miR-497a-5p	− 2.2768
mmu-miR-186-5p	− 2.0988
mmu-miR-15a-5p	− 2.0609
mmu-miR-17-5p	− 2.0469

### Analysis of miRs Expression Profile During the Course of EAE

**Fig. 3:**
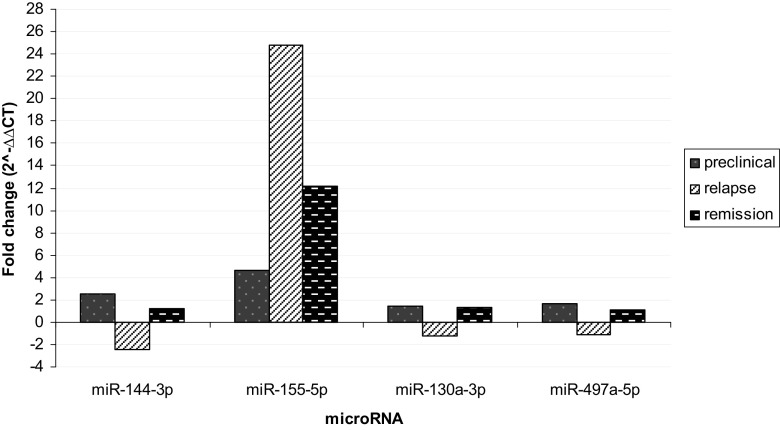
Relative expression level of microRNAs during the course of EAE. RNA isolated from the brain of mice at various clinical stages were used. Results from all disease phases (preclinical, relapse, remission) were refered to results obtained from healthy mice (control) and presented as a fold change (2^−∆∆CT^)

Changes in expression profile of seven deregulated microRNAs (miR-302d-3p, miR-291a-3p, miR-694, miR-144-3p, miR-155-5p, miR-130a-3p, miR-497a-5p) were studied during various disease phases: late preclinical phase (to obtain visible differences between this disease stage and healthy animals), relapse, and remission, compared to healthy controls (*n* = 10 for all groups). For miR-694 and miR-291a-3p, the threshold cycle (CT) data were almost at the cutoff value (CT ~ 36), whereas miR-302d-3p was very unstable during our experiments (data not shown). For these reasons, they were excluded from further analysis. The most upregulated microRNA in all disease stages was miR-155-5p with fold change 4.7; 24.8; 12.1 in preclinical phase, relapse, and remission, respectively. Also, miR-144-3p reaches the significant fold change in our study (2.5 in preclinical phase and – 2.4 in relapse). miR-130a-3p and miR-497a-5p did not show any relevant changes in their expression level at any disease phase (Fig. 3).

### IL-17 Alters the Expression Level of miR-155-5p and miR-144-3p in Preclinical EAE Mice, but Not in Healthy Animals

**Fig. 4:**
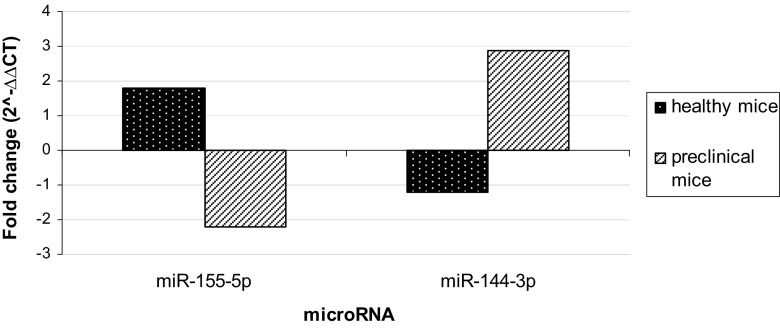
microRNA expression level after stereotactic brain microinjections of IL-17 in healthy mice and animals in preclinical phase of EAE. RNAs isolated from brain homogenates were used (see the “Materials and Methods” section). Results obtained for animals after intracerebral injection of IL-17 were referred to the results from animals after PBS microinjections (control), and presented as a fold change (2^−∆∆CT^)

Two microRNAs with potential role in EAE pathogenesis—miR-155-5p and miR-144-3p—were analyzed in mice during the preclinical phase of EAE (5 days post immunization) and in healthy animals. Both groups were subjected to stereotactic brain microinjections of 100 ng IL-17 or PBS (control) to further confirm the relationship between the studied non-coding RNAs and IL-17 (*n* = 10 for each group). Obtained results showed that high concentration of this cytokine was able to downregulate miR-155-5p (fold change − 2.2) and to upregulate miR-144-3p (fold change 2.9) in preclinical animals. We did not observe any significant changes in microRNA expression in healthy animals after brain microinjections with IL-17. Such observation suggests that this cytokine is not a direct regulator of studied miRs and that other factors related to pathologic processes are responsible, at least in part, for such miR deregulation (Fig. 4).

### Computational Analysis of miR-155-5p Target Genes

**Fig. 5:**
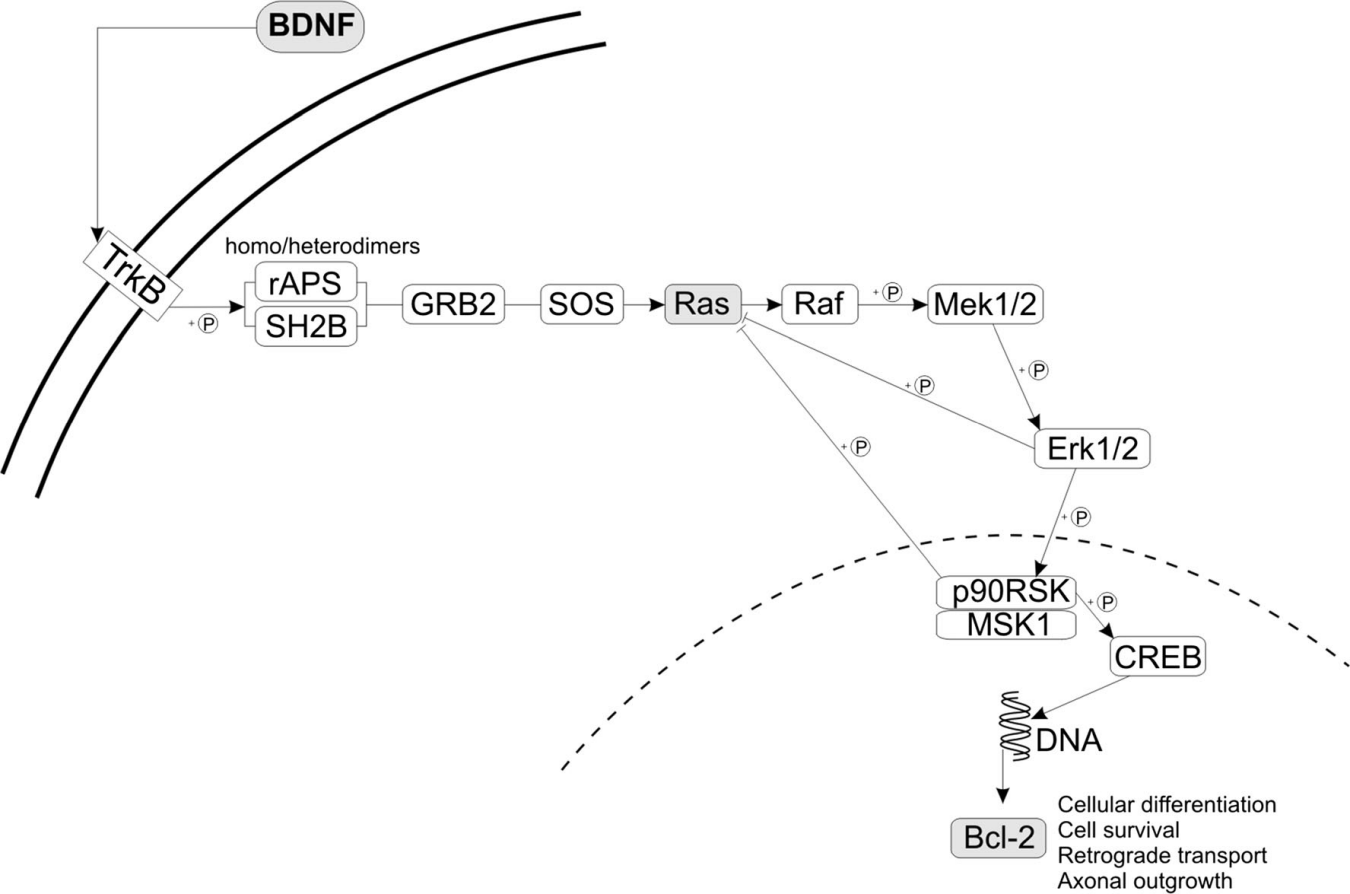
Computational analysis of miR-155-5p target mRNAs and altered pathway—neurotrophin signaling pathway. Targets analyzed in our study are marked in gray squares (graph based on the KEGG pathway mmu04722 (Kanehisa and Goto 2000; Kanehisa et al. 2016))

The miR-155-5p is a well-known molecule regulating various aspects of EAE and MS (see the “Discussion” section). In our study, we have observed that IL-17 is able to downregulate the expression level of miR-155-5p during preclinical phase of EAE. As this is an unexpected result, we decided to further analyze only this miR. To identify cellular pathways and mRNA targets that may be altered by decreased expression of miR-155-5p, we used online databases, such as DIANA mirPath v3.0, TargetScan Mouse Release 7.1, and miRecords (last updated on April 27, 2013). For further analysis, we chose neurotrophin signaling pathway (based on DIANA mirPath). Among the genes from this pathway, we chose *BDNF* (positive results from miRecords and TargetScan databases, also experimentally validated as a target gene for miR-155-5p (Varendi et al. 2014)) and *BCL2* (positive results from DIANA mirPath and miRecords databases) (Fig. 5). Additionally, we studied expression alterations in *HRAS* gene participating in the same pathway.

### IL-17 Induces Elevated Expression of *BDNF*, *HRAS*, and *BCL2*

**Fig. 6:**
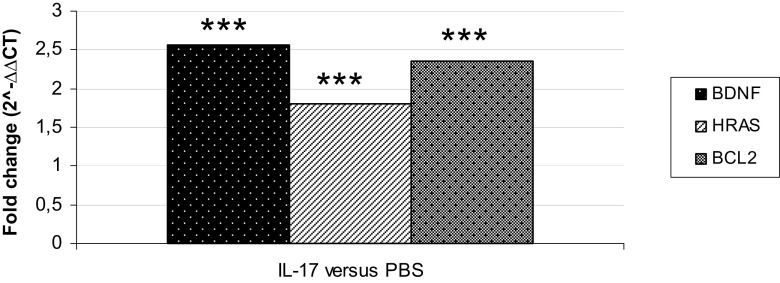
mRNA expression level after stereotactic brain microinjections of IL-17 to mice in preclinical phase of EAE. RNAs isolated from brain homogenates were used (see the “Materials and Methods” section). Results obtained for animals after intracerebral injection of IL-17 were referred to the results obtained for animals after PBS microinjections (control), and presented as a fold change (2^−∆∆CT^) ***p < 0.001

To assess the impact of high-dose IL-17 on target genes (*BDNF*, *HRAS*, *BCL2*), we prepared real-time PCR analysis on RNA isolated from mice during preclinical phase of EAE (5 days post immunization) after brain microinjections of IL-17 or PBS (control) (*n* = 10 for each group). All studied mRNAs were significantly increased after intracerebral injection of IL-17 compared to those of control group (*BDNF*—fold change 2.6, *p* = 0.0007; *HRAS*—fold change 1.8, *p* = 0.0002; *BCL2*—fold change 2.3, *p* = 0.0006) (Fig. 6).

### Expression Level of *BDNF* and *BCL2* mRNA Correlates Negatively with the miR-155-5p Level in Preclinical Animals

**Fig. 7:**
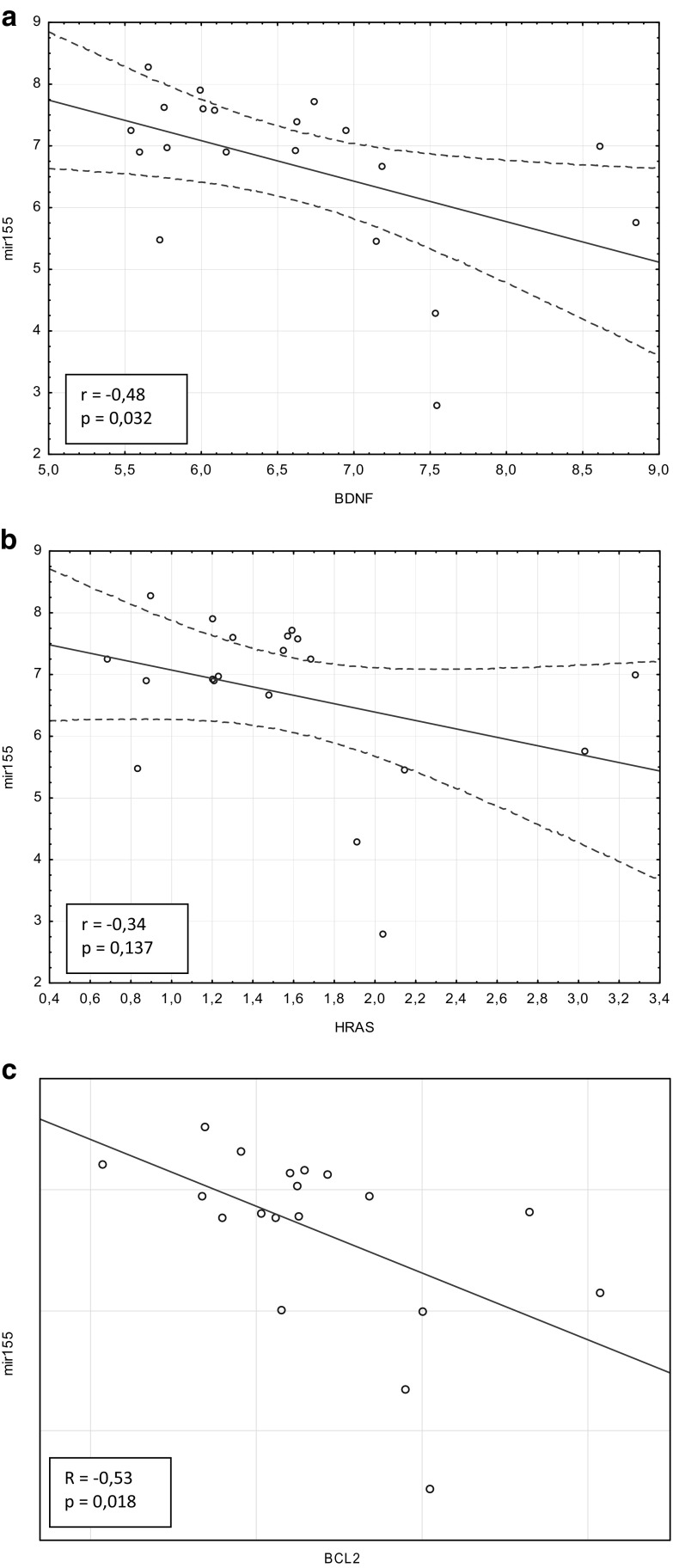
Analysis of correlation between miR-155-5p and mRNA for: *BDNF* (**a**), *HRAS* (**b**), and *BCL2* (**c**). Analysis was performed for results obtained from mice in preclinical phase of disease, after intracerebral injections of IL-17 or PBS (control). r Pearson’s correlation coefficient, R Spearman’s correlation coefficient

To assess the correlation between the expression level of miR-155-5p and genes of interest, we have analyzed results obtained from the CNS of preclinical mice after IL-17 or PBS microinjections. *BDNF* and *BCL2* mRNA correlated negatively with miR-155-5p expression level (Pearson’s correlation, *r* = − 0.48 and *p* = 0.032 for *BDNF*; Spearman’s correlation, *R* = − 0.535 and *p* = 0.018 for *BCL2*). No correlation was observed between *HRAS* and miR-155 (Pearson’s correlation, *r* = − 0.344 and *p* = 0.137) (Fig. 7a–c).

### IL-17 Upregulates HRas and Bcl-2 Protein Level, but Not BDNF

**Fig. 8:**
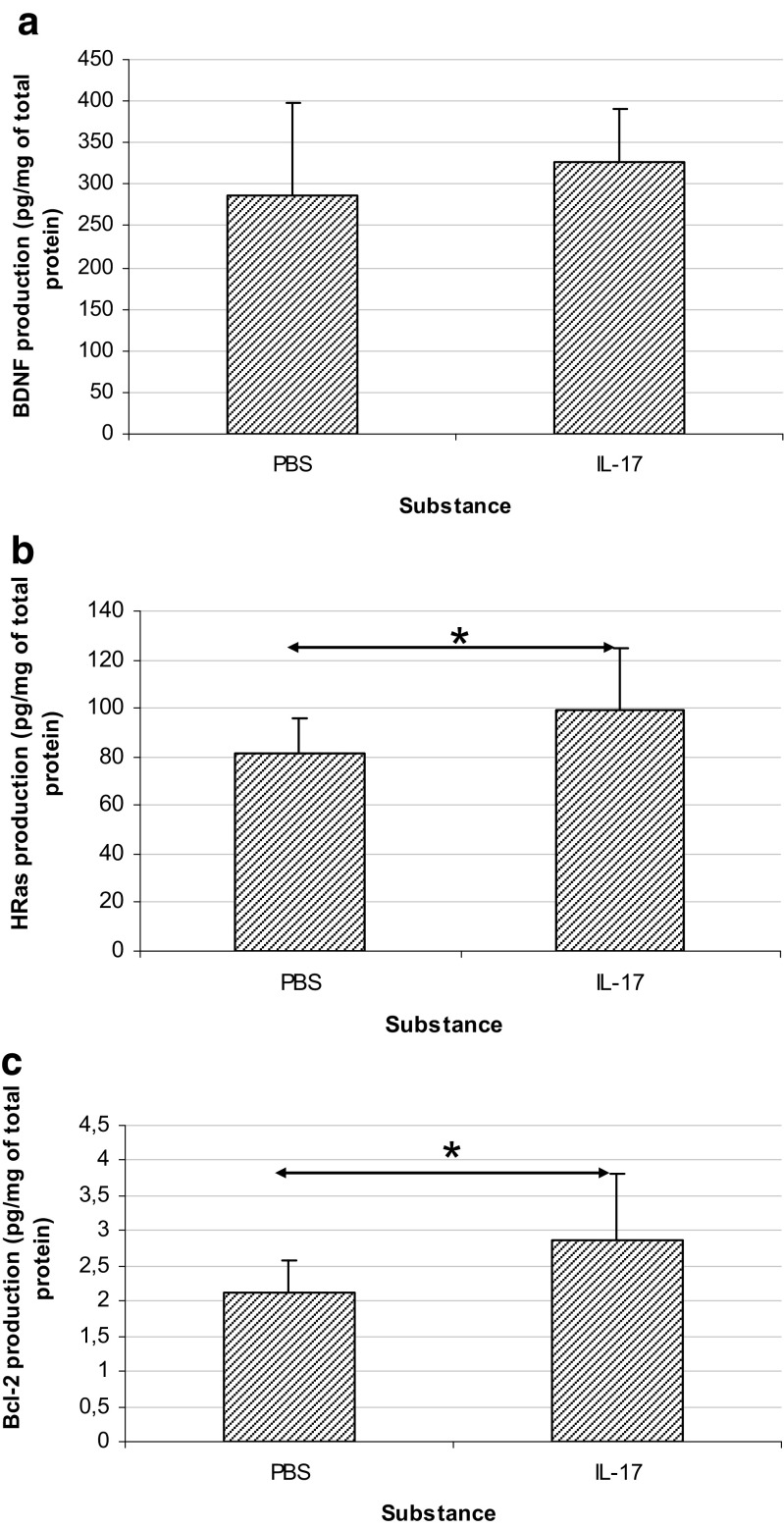
The protein level studied in the homogenates of brains from preclinical EAE mice after intracerebral injection of IL-17 or PBS (control)—BDNF (**a**); HRas (**b**); Bcl-2 (**c**) expression. Data are presented as mean ± SD; *p < 0.05

To further analyze the impact of IL-17 on studied cellular pathway, we performed ELISA tests for selected proteins (*n* = 10 for each group). We have observed statistically significant increase in HRas (*p* = 0.032) and Bcl-2 (*p* = 0.036) protein expression in preclinical mice (5 days post immunization) after IL-17 brain injections (Fig. 8b, c), similarly to what was observed in their mRNA analysis. However, in contradiction to the results obtained from mRNA experiments, we did not observe any statistically significant changes in BDNF protein level in our experimental conditions (*p* = 0.333) (Fig. 8a).

## Discussion

Multiple sclerosis (MS) is an inflammatory disease, characterized by pronounced immune cells infiltration into the central nervous system (CNS). Activated inflammatory cells secrete wide variety of factors, leading to blood-brain barrier (BBB) disruption, degradation of myelin, and neuronal damage (Popescu and Lucchinetti 2012). It is believed that interaction between environmental factors, and genetic and epigenetic alterations play important roles in MS development. One of the epigenetic factors implicated in MS pathology is microRNAs (miRNAs, miRs). miRs are universal post-transcriptional modulators, as one miR may bind to multiple target mRNAs, and single mRNA may be regulated by various miRs. As miRNA may possess numerous binding sites within target mRNA, even subtle expression alteration of miR may exert profound effect on protein expression level. Increasing data suggest the involvement of miRs in the process of autoimmunity and neuroinflammation (O’Connell et al. 2010b). In this study, we have analyzed the expression profile of miRs related to these processes in the context of EAE.

There are numerous studies describing the role of microRNAs in the pathogenesis of MS and EAE (Ksiazek-Winiarek et al. 2013; Ma et al. 2014). miR-155 is one of the widely studied and the most characterized small non-coding RNAs. Its regulatory role has been described for numerous cell types. It was shown that this molecule promotes differentiation of Th17 cells, inhibits Th2-type immune response, and enhances activation of T cells, mononuclear phagocytes, and dendritic cells (DCs), thus leading to escalated inflammatory process (Dunand-Sauthier et al. 2011; O’Connell et al. 2010a; O’Connell et al. 2007). It was observed that miR-155 also supports re-activation of encephalitogenic CD4+ T cells (Jevtić et al. 2015). What is more, it affects also the CNS resident cells. It regulates inflammatory activity of microglia, while it inhibits anti-inflammatory role of astrocytes (Cardoso et al. 2012; Junker et al. 2009). miR-155 was also indicated as one of the most upregulated miRs in acute MS lesions (Junker et al. 2009). Among all molecules analyzed in our study, miR-155-5p was the most upregulated in all disease phases.

The important role of miR-155 in the development of EAE was also confirmed by the studies utilizing various miR-155-knockdown animals. miR-155−/− mice had significantly reduced number of Th17 cells and were partially resistant for EAE development (Murugaiyan et al. 2011; O’Connell et al. 2010a). It was concluded that these observations are consequence of the altered responses of T helper cells in the CNS and peripheral lymphoid organs (Murugaiyan et al. 2011). These results are in agreement with our present and previous findings (Wojkowska et al. 2014). In present study, downregulated expression of miR-155 was observed after intracerebral injection of IL-17 in preclinical EAE mice. It was shown in our previous study that in the same experimental conditions after IL-17 administration, there was reduced accumulation of Th17 cells in the brains of preclinical mice (Wojkowska et al. 2014). Altogether, our results further support the relation between IL-17, miR-155, and Th17 cells in the EAE pathogenesis. Moreover, results obtained in this study indicated potential protective function of high-dose IL-17 via miR-155 deregulation. To get further insight into the cellular processes affected by decreased miR-155 level, we have prepared in silico analysis. Based on results from three databases (TargetScan, miRecords, and DIANA mirPath), we have concentrated on the neurotrophin signaling pathway, and we have selected *BDNF*, *HRAS*, and *BCL2* genes for additional analyses in the context of preclinical stage of EAE.

BDNF is a neurotrophin essential for development and maintenance of the nervous system (Thoenen 1995). Mature BDNF binds to two different types of receptors: high-affinity tyrosine kinase receptor B (TrkB), and low-affinity p75NTR receptor (Hempstead 2002). Previously thought to be express mainly in the nervous system, recent studies have indicated that BDNF is also produced by various subpopulations of immune cells (Edling et al. 2004). It was shown that BDNF is present in MS lesions, but is also expressed in peripheral blood (Kerschensteiner et al. 1999; Stadelmann et al. 2002). Furthermore, De Santi et al. have shown that T cell subpopulations not only release BDNF, but also express the full-length TrkB receptor, indicating that they may be regulated in the paracrine or autocrine manner (2009). BDNF via TrkB may regulate cell apoptosis. Makar et al. have shown that this process was significantly enhanced in the brain and spinal cord of EAE mice (2008). Treatment with BDNF reduced the severity of EAE, and the authors indicated that this effect was related to the reduction of apoptosis observed both in the brain and spinal cord. Moreover, Almaida et al. indicated that in vitro treatment of hippocampal neurons with BDNF reduced their sensitivity to glutamate-induced apoptosis, partly via upregulation of Bcl-2 (2005).

The anti-apoptotic function of BDNF and its expression by immune cells may result not only in neuronal cells protection, but also in prolonged T cells’ survival (De Santi et al. 2009). Indeed, in the studies utilizing chronic experimental EAE mice with BDNF gene deletion from the T cell lineage and myeloid cells, diminished inflammatory response and limited neuroinflammation were observed (Linker et al. 2010).

It has been noticed that BDNF expression is regulated by cytokines released from immune cells during EAE and MS. Cytokines produced by Th2 cells upregulate the BDNF gene expression in glial cells in vitro, whereas Th1 cytokines downregulate the expression of genes for both BDNF and its receptors (Lisak et al. 2006, 2007). Our study shows that Th17-related cytokine—IL-17—may influence the BDNF expression level. Here, we have observed increased expression of BDNF gene during preclinical phase of EAE following intracerebral administration of IL-17. As we have analyzed whole brain homogenates, it is difficult to state in what type of cells increased expression of BDNF mRNA was present. In our study, we have analyzed BDNF 1 mRNA, which represents the longest transcript and which encodes the longest protein (NCBI, accession NM_007540). Kruse et al. have shown that in contrast to the brain, where all transcript variants have been observed, immune cells and peripheral lymphoid organs have expressed only BDNF 3 mRNA (2007). Even after T cell activation, only BDNF 3 mRNA has been upregulated (Kruse et al. 2007). Liu et al. in their study have obtained different results, as they have shown the presence of BDNF 3, 4, and 5 mRNA in the spleen (2006). Based on these results, we concluded that elevated levels of BDNF mRNA after intracerebral injection of IL-17 relate to neuronal resident cells, rather than immune infiltrating cells. However, this needs further experimental confirmation.

Although we have observed elevated levels of BDNF mRNA in our experimental conditions, there were no significant changes in the protein level. It was experimentally validated that miR-155 directly represses BDNF through binding to its predicted site in BDNF 3′UTR. Variants of BDNF mRNA transcripts may differ in the length of their 3′UTR and it was shown that only BDNF variant with long 3′UTR contains the binding site for miR-155 (Varendi et al. 2014). Moreover, the relative expression of various 3′UTR isoforms of BDNF varies among different brain regions, suggesting that they may possess different biological functions (An et al. 2008). We did not analyze the specific brain’s region, but rather whole organ, thus what we have observed in our study is an averaged change in BDNF level, and this may be the reason why we have not obtained significant alteration in the content of this neurotrophin. As it is more evident that the exact role of BDNF is complex and context-dependent, our results may also suggest the tight regulation of BDNF expression on the translational level. The elevated production of BDNF may lead to more pronounced T cell infiltration into the CNS, increased cytokine production, what contributes to the exacerbated disease process. The lack of increase in BDNF protein expression in our study, despite of elevated mRNA levels, may indicate the presence of regulatory mechanisms that prevent the harmful BDNF action related to prolonged immune cells’ survival and enhanced neuroinflammatory process.

As it was mentioned earlier, BDNF may act as an anti-apoptotic factor through the regulation of the Bcl-2 expression. Indeed, in our experiments, we have observed upregulation of Bcl-2, both on mRNA and protein level. Members of the Bcl-2 protein family are key regulators of the intrinsic (“mitochondrial”) apoptosis pathway (Antonsson 2004). Previous studies suggested that elevated levels of Bcl-2, an anti-apoptotic protein, protect hippocampal neurons from glutamate-mediated excitotoxicity, and Bcl-2 or other close family members may participate in neurotrophin-dependent survival (Deshmukh and Johnson 1997; Michaelidis et al. 1996; Wong et al. 2005). One of the major regulators of Bcl-2 transcription is cAMP-responsive element-binding protein (CREB), which was also shown to mediate neurotrophin-induced transcription (Finkbeiner et al. 1997). This suggests that CREB regulates expression of genes implicated in the prosurvival action of neurotrophins (Bonni et al. 1999).

Proteins belonging to the Ras family are small GTPases, participating in the regulation of numerous cellular processes ranging from cellular growth, differentiation, survival to chemotaxis, transport, and apoptosis (Karnoub and Weinberg 2008). One of the Ras isoforms, highly expressed in the CNS, is HRas. It is known for many years that HRas can stimulate cellular cycling, differentiation, or survival (Cox and Der 2010). However, relatively recent studies have indicated that HRas can also induce cell apoptosis, depending on the balance between various downstream effector molecules (Cox and Der 2003, 2010; Xue et al. 2000) Bcl-2 was shown to inhibit Ras-induced apoptosis. It was observed that HRas and Bcl-2 may interact with each other under physiological conditions, and that this interaction is even enhanced in a pro-apoptotic environment (Chen and Faller 1996). Moreover, the presence of activated Ras increases Bcl-2 activation via its phosphorylation, further increasing their association. It means that downregulation of Bcl-2 phosphorylation decreases its interaction with HRas, making cells more susceptible to apoptosis (Chen and Faller 1996). This has suggested that in the presence of apoptotic stimuli, Bcl-2 interaction with HRas has partly neutralized Ras pro-apoptotic signal (Denis et al. 2003).

In our study, we have analyzed the alterations in HRas expression level after IL-17 administration during initial stage of EAE. We have found significant increase both in mRNA and protein levels. As was suggested by Chen and Faller, elevated expression of HRas may result in more pronounced activation of Bcl-2 and protection from apoptosis (1996). Additionally, it was shown that activation of HRas downstream protein kinases may result in induction of several transcription factors, like c-Fos or CREB, resulting in synaptic plasticity and survival (Stacey et al. 1987; Vanhoutte et al. 1999). This further supports the relationship between BDNF, HRas, and Bcl-2 analyzed here.

Results obtained in our study suggest the protective role of IL-17 in the initial phase of EAE. This was mediated by the downregulation of pro-inflammatory microRNA—miR-155-5p—and subsequent upregulation of its target mRNAs. The elevated expression level of HRas and Bcl-2 suggested anti-apoptotic function of high dose of IL-17 in our experiments. There are evidences that IL-17 is not exclusively a pro-inflammatory factor, and that its role depend on the specific environment present during inflammation. Hu et al. have shown in their study that IL-17 may exert anti-apoptotic effect during inflammation (2014). Supernatants obtained from stimulated astrocytes especially those with high concentration of IL-17 have protected cultured neurons from apoptosis. It was also noted that astrocytes treated with IL-17 alone (without inflammatory media) were not able to produce sufficient amounts of IL-17 to protect primary cultured neurons (Hu et al. 2014). Indeed, we have seen the effect of IL-17 on miR-155-5p expression level in preclinical phase of EAE, but not in healthy mice, indicating that other factors related to the process of inflammation are necessary for IL-17 to exert its protective function. The anti-inflammatory potential of IL-17 has been also observed in study utilizing experimental autoimmune uveitis (EAU) in mice. Animals subjected to the IL-17 administration unexpectedly demonstrated delayed onset of the disease and ameliorated clinical signs. This was attributed to the altered function of autoreactive T cells, as their proliferation, autoreactive response, and cytokine production were diminished. It was shown that T cells isolated from IL-17-treated animals have a suppressor activity on EAU development, as an adoptive transfer of these cells did not result in disease induction in susceptible animals (Ke et al. 2009). This protective effect of IL-17 was observed only if the cytokine injections were done during the initial phase of EAU. IL-17 administration at the disease onset did not induce any significant alterations in disease course. If this is also true in our experimental conditions, it needs further investigations, as we have studied here the effect of IL-17 intracerebral injections during the early preclinical phase of EAE. The impact of IL-17 administration during relapse and remission phase of disease will be studied in future.

Contradictory results have been observed in glomerulonephritis mouse model (Hamour et al. 2015), where protective function of IL-17 was observed in acute phase of disease. These results indicated the importance of specific features characteristic for various inflammatory disorders, influencing the time point at which IL-17 may exert protective role.

Studies utilizing cell cultures, animal models, or samples from patients give strong justification for therapeutical targeting of IL-17 in human inflammatory disorders, with an aim to alleviate harmful and often painful manifestation of disease. In light of the recent results concerning the protective function of IL-17 in autoimmune, inflammatory diseases, this therapeutical approach should be rethought and taken into consideration with caution.

Results from our study indicated the protective role of IL-17 in the initial phase of EAE. This effect was related to the downregulation of pro-inflammatory microRNA—miR-155-5p. Moreover, we have proposed the potential mechanism responsible for the anti-apoptotic action of IL-17. We have identified elevated expression level of *BDNF*, *HRAS*, and *BCL2* mRNAs, all participating in the pro-survival pathway. We have also conducted studies on the role of CXCL-1 administration in preclinical animals in the same experimental design. We have not found any significant alterations in miR-155-5p level in initial disease phase, nor in BDNF, HRas, and Bcl2 level (data not shown). Such observations further support the conclusion that observed results were specific to IL-17A. As for our knowledge, this is the first study linking protective function of IL-17 with altered miR expression levels in the animal model of multiple sclerosis.

The main limitation of our study is that the whole brain homogenates were analyzed, and therefore it is not directly indicated what cell types are protected from apoptosis, as in the early preclinical phase of disease, the migrating immune cells are already present (Wojkowska et al. 2014).
